# Student perceptions of the care of children: impacts of pre-clerkship pediatric and primary care clinical teaching

**Published:** 2014-12-17

**Authors:** Beverley Karras, Saumya Selvaraj, Athena McConnell, Deirdre Andres, Krista Trinder, Meredith McKague

**Affiliations:** 1 Department of Academic Family Medicine, University of Saskatchewan, Saskatchewan, Canada; 2 Department of Family Medicine, University of Calgary, Alberta, Canada; 3 Department of Pediatrics, University of Saskatchewan, Saskatchewan, Canada; 4 College of Medicine, University of Saskatchewan, Saskatchewan, Canada

## Abstract

**Background:**

Pediatric clinical skills teaching sessions provide an early opportunity for students to be exposed to the medical care of children. This report describes second and third year medical students’ perceptions of and attitudes towards working with children before and after the pediatric clinical skills teaching sessions, and the experiences of those students precepted by pediatricians only compared to those students working with a combination of pediatricians and family physicians.

**Method:**

A 13 question survey was voluntarily completed before and after teaching sessions. Written reflective assignments were qualitatively analyzed for key themes. Response rate averaged 68% with class sizes of 84 and 85 students.

**Results:**

Students’ perceptions of the care of children were generally very positive. Some differences were found based on gender, phase of study and prior clinical exposure to pediatric care. Pre and post responses were similar, regardless of preceptor specialty. Students with family physician preceptors identified the themes of prevention, health promotion and multidisciplinary care in their reflections.

**Conclusions:**

Students had already formed positive attitudes toward the medical care of children and intended to care for children in their future practice. Further research is needed into the effects of pre-clerkship experiences in the care of children on choice of medical specialty.

## Introduction

In pediatrics, the skills of history taking and physical examination differ from those used with adults. Most Canadian medical schools teach these unique clinical skills in specific sessions, which also provide an early opportunity for medical students to be involved in the care of children.

Published literature has revealed that medical students consider many factors in their decision to pursue different careers, including lifestyle and income expectations.^1^–^4^ Gender also appears to play a role, with surveys of trainees showing greater tendencies for women to choose family medicine or pediatrics.^2^,^5^ To date there has been little published on the role of early clinical exposure in the career decision making process. A 2001 study of students matched to family medicine residencies found that meaningful exposures in pre-clinical years was a major influence in choosing this post-graduate area,^6^ although an earlier synthesis of the literature did not find this association.^2^ A University of Toronto study revealed that a pre-clerkship observership led to a change in attitudes and increased interest towards Emergency Medicine.^7^ The effect of pediatric pre-clerkship exposure towards subsequent interest in and attitudes about working with children has not previously been studied.

Traditionally, pediatricians have taught the pre-clerkship sessions in our institution. The Future of Medical Education in Canada Report has challenged medical schools to include a generalist perspective and to involve generalist physicians at all stages of undergraduate medicine.^8^ At the University of Saskatchewan, in 2010–11, pediatric and family medicine faculty collaborated to teach these skills to third year students, while the same skills were taught to second year students by pediatricians alone. Pediatricians taught either in hospital or community settings, while family physicians taught exclusively in community settings.

The changes to the curriculum were evaluated using a mixed methodology. We sought to answer the following questions:

1. What were students’ perceptions of, and attitudes towards, the medical care of children before and after these sessions?
2. What effect did the addition of family medicine preceptors have on students’ perceptions of, and attitudes towards, the medical care of children?

The Ethics Board of the University of Saskatchewan approved the research questions and methodology.

## Methods

### Study design

Methodology included a cohort attitudinal survey completed by students (Appendix A), and analysis of guided reflective essays (Appendix B). Students had previous practice in writing reflective assignments prior to this learning experience.

A 13-question survey included demographics, previous clinical experience with children, and asked respondents to list determinants of health for Saskatchewan children. Questions also assessed medical students’ attitudes and beliefs regarding the medical care of children using a five-point Likert scale. As no relevant validated survey was found in the literature, items were drafted by two of the authors based on the research questions and pre-tested by the other authors and reviewed for face validity. The survey was completed by three final year medical students who provided feedback. Based on this, the survey was further modified, and it was made available for voluntary online completion. It was completed before and after the clinical skills teaching blocks. Surveys were completed within six weeks of finishing the teaching sessions.

Details of the approach used to the qualitative component of the study are described in the Analysis section.

### Setting

This English-language medical school in western Canada includes a four-year undergraduate medical program, with a two and a half-year pre-clerkship and a one and a half-year clerkship component. At the time of this study, the first and second years of the MD program were only offered at one program site (Saskatoon), while the final term of pre-clerkship for third year students and clerkship were offered at two program sites (Regina and Saskatoon).

The medical school was undergoing expansion in student numbers at the time of this study. Due to curricular changes, both second and third year students received pediatric clinical skills sessions during the study year.

### Sample size and sampling methods

All 84 second year students and all 85 third year students were invited to participate in the pre- and post-sessions surveys.

All third year students were required to submit a reflective essay following their teaching sessions. An administrative staff member randomly selected 10 of 85 anonymized assignments for qualitative analysis. Another four were subsequently selected and analyzed to enhance representation of students who had worked with family medicine tutors.

### Outcome measures

Outcome measures included student responses to survey items regarding their perceptions of, and attitudes toward, the care of children. For the qualitative portion of the study, outcomes were the themes regarding pediatric care identified by students within the reflective assignment.

### Analysis

Quantitative statistical analyses of cohort responses before and after clinical sessions were conducted using SPSS Statistics 19. Analyses included t-tests, ANOVAs and chi-squares. Effect sizes (Cohen’s d) were calculated to determine practical significance, where 0.2 is small, 0.5 is medium, and 0.8 is large. As responses to most items were negatively skewed, reflections and log10 transformations were applied. Results were similar for analyses conducted on raw and transformed data so only analyses on raw data are reported.

Reflective assignments were read and coded for themes by one author (SS), with review by the other authors. Assignments were read in an iterative process for themes, using a process of constant comparison. Themes were then added together, subdivided or collapsed based on commonality between topics. Qualitative analysis continued until saturation was achieved.

## Results

The overall student response rate (combined pre-and post-survey) was 68.6%. The surveys were conducted using a cohort design, with approximately 75% of the students who responded to the pre-teaching survey also responding to the post-teaching survey. Second year students were all training in the same city (Saskatoon). Third year students were training at one of two program sites (Saskatoon, Regina).

Detailed response rates and demographics are found in Table 1.

The majority of students who completed the survey were younger than 25, identified as women, were not parents, and did not have prior pediatric experience. Detailed demographic information is found in Table 1. There were no statistically significant differences in demographics between pre and post survey respondents. Thus, the students who completed the post-test were comparable to those who completed the pre-test in these areas.

Types of previous pediatric clinical exposure included observerships, paid summer externships, and volunteer experiences. Students with previous exposure described having worked with or observed family physicians, pediatricians, nurse practitioners, public health nurses and emergency room nurses involved in the clinical care of children.

### Attitudes toward the care of children

Students’ responses to questions about their perceptions of the care of children were generally very positive and are summarized in Table 2. Students in all groups indicated that they were likely or highly likely to provide medical care to infants/children in their future practice. The majority agreed or strongly agreed that the care of children is valued by the medical profession and society.

Students showed low rates of agreement with the statement that income is an incentive to consider providing pediatric care. The majority agreed that nurse practitioners, pediatricians and family physicians all played significant roles in the care of well children.

### Pre- and post-survey

Few significant differences were found between pre and post survey cohort results. The exception was that second year students were significantly more likely to agree with the statement “Care of children is valued in the medical profession” before attending sessions than after attending sessions (*t*(130) = 2.30, *p* = .023, *d* = .40). (See Table 2).

### Preceptor type

The effect of preceptor specialty was analyzed using data from third year groups in Saskatoon only, due to small numbers in other groups. There were no significant differences on prior clinical experience with children or responses to attitudinal questions between students taught by the different preceptor types.

### Prior clinical experiences with children

Overall, no significant differences were found between students who had prior clinical experiences with children and those who did not. While no significant differences were noted among second year students, third year students in Saskatoon with prior clinical exposure were more likely to feel that care of children is valued within society on the pre-survey (M = 4.86, SD = 0.36) than those without prior exposure (M = 4.46, SD = 0.59; t(43) = −2.70, p = 0.010). As well, on the post-survey, 3rd year students in Saskatoon were more likely to agree that there was a role for pediatricians in the care of well children if they had prior clinical exposure (M = 4.86, SD = 0.35 vs. M = 4.43, SD = 0.90; t(43) = −2.10, p = 0.042).

### Student group

A statistically significant difference between student groups was found on the pre-survey for the question asking whether care for children was valued within the medical community (*F*(2,119) = 11.18, *p* < .001). Post-hoc analyses indicated that second year students gave a significantly higher response to this question than third year students at either site (see Table 2). Statistically significant differences by student group were not found for any other items.

### Students’ gender and age

Statistically significant gender differences were not found for second year students. In the pre-survey, third year women were more likely than men to indicate an intention to provide care for infants/children and that there was a significant role for nurse practitioners in the care of children (see Table 3). However, these differences were not found on the post-survey. On the post-survey, third year women were more likely than men to report a significant role for family physicians in the care of children. Age was not a significant factor.

### Qualitative analysis results

A common theme that emerged in student assignments is the **uniqueness of pediatric medicine**. Three related sub-themes were identified: the **skills used to assess and care** for children**,** the **relationship triad** (patient, caregivers, and physician), and the importance of **addressing holistically the health determinants** affecting children.

Students described several **skills** important in the care of children. Many identified the ability to remain calm and patient as an asset. As one student learned, “*creativity in your approach to a physical exam and history of a pediatric patient was essential*.” Students identified methods of integrating play into various parts of the physical exam: “*bouncy balls and toy cars are great mediums (sic) to watch gait*.”

Some students commented that, due to limited communication skills in small children, health care providers must utilize a variety of sources of information to assess a child’s condition and needs. Students identified the need for physicians to be opportunistic and flexible.

Factors such as crying, short attention spans, and poor compliance force the examiner to perform certain parts of the exam whenever they can, making the pediatric exam much less ordered.

No two children are the same, and so each pediatric exam is an adventure in building rapport with a child and figuring out ways to get them to cooperate as much as possible.

Students emphasized the importance of the **relationship triad** between the child, caregiver(s) and physician in pediatric care**.** One student reported, “*physicians who work in pediatrics work with families as much as with children*.” When addressing the medical care of children, students noted the benefits of a trusting relationship between the physician and the child’s parents/caregivers.

If their child is sick, the majority of parents will be frightened and need a physician who is attuned to this; someone who will take the extra time to answer their questions and provide reassurance where appropriate.

Students noted that a **holistic approach that addressed determinants of health** was needed to manage children’s health issues. Multidisciplinary care was identified as being important in order to address the wide range of health needs and to focus on prevention, health promotion and counseling. Determinants of health identified by students included diet, activity level, immunizations, socioeconomic status, home environment, social environment, family factors, gender, culture, ethnicity, health care access and access to health information.

Most treatment plans assume the patient has a nice place to live and rest, money for medications, access to a nutritional diet, and many other things that are not afforded by the thousands living in poverty in our city alone.

Students taught by family physicians in a primary care centre identified multidisciplinary care within a single facility as valuable to patients. Students in primary care settings also noted a connection existing between a child’s health and parental and family factors, and the importance of addressing these determinants of health within preventative care. Similar themes did not appear in the reflective assignments of students taught by pediatricians.

The consults I participated in and observed taught me the importance of establishing a sense of the child’s context at home, at school, and socially. This exercise helps to identify health concerns specific to the patient’s situation and understand what role the physician should play in health promotion for that child.

## Discussion

Our study found that medical students demonstrated positive attitudes toward the care of children generally. Although attitudes were positive in all surveys, we noted that fewer second year students agreed that the care of children is valued by the medical profession after their teaching sessions than before their sessions. The reflective essays did not provide an explanation for this apparent change in attitude. It is unclear whether this is a direct result of the pediatric clinical sessions, other experiences occurring during their second year including possibly the influence of the hidden curriculum,^9^ or if there was an initial overestimation by students with less clinical experience. We note that the second year students’ responses to this question were higher than the third years’. In addition to the factors mentioned above, this may represent cohort differences between the two classes. Further inquiry into this phenomenon, especially qualitative research to delve into potential mechanisms, is certainly warranted.

Many factors influence medical students’ decisions regarding which residency programs to pursue;^1^–^4^ early exposure may be one important factor. There is limited literature assessing the effect of pediatric pre-clerkship exposure towards interest in and attitudes about working with children. In our study, following pediatric clinical skills sessions, student attitudes remained very positive. However, our study was not designed to evaluate impact on eventual career choice. Further research in this area is required.

Income expectations are one influence on career choice, and may be a more important factor for those selecting non-primary care careers.^2^,^3^ Students in our study reported that income is a disincentive to providing pediatric care. Despite this, the majority indicated an interest in providing pediatric care in their future practice. The wording of our question did not allow us to determine whether students interpreted this to mean careers in general pediatrics and family medicine or if this included higher income subspecialty careers (e.g. pediatric general surgery).

The gender difference among third year students on the pre-survey is consistent with existing literature that women are more likely to become family physicians or pediatricians.^2^,^5^ This difference was no longer significant on the post-survey. This may have been due to differences in clinical experiences for both genders.

The integration of family medicine preceptors into pediatric clinical skills teaching was a new initiative. Survey results demonstrated no significant difference in the interest and attitudes toward the medical care of children when students received some of their teaching from family physician preceptors. In the reflective assignments, students who had exposure to family medicine preceptors commented on the importance of multidisciplinary and preventative care, as well as family influences on child health. This may reflect preceptor discipline, but may also reflect teaching location (community site vs. hospital). There appear to be no disadvantages and some unexpected advantages to pre-clinical instruction by family preceptors working in the community.

This study found that medical students expressed a high level of interest in the care of children prior to the pre-clerkship clinical skills sessions and this high level of interest was minimally affected by their clinical experience. This may suggest the possibility of a ceiling effect, our inability to detect differences, or both.

Students identified several unique aspects of providing care for children including a particular skill set, the importance of a patient and family centered clinical method, and addressing determinants of health. This finding supports the need for designated pediatric clinical skills teaching by preceptors with experience caring for children.

Study limitations included: fewer third year students responded to the surveys (however those who did respond were representative of the class); pre and post survey respondents were not linked and therefore the differences noted do not reflect individual changes; only 75% of the students who answered the pre-survey also answered the post-survey; and since there were no groups taught solely by family physicians, our ability to identify differences between preceptor types may have been limited.

### Conclusion

These results indicate that second and third year medical students have already formed positive attitudes toward the medical care of children and intend to care for children in their future practice. The involvement of family physicians in teaching pre-clerkship pediatric clinical skills is supported by this study.

Further research is needed into the effects of pre-clerkship experiences in the care of children on choice of medical specialty.

## Figures and Tables

**Table 1:** Survey demographics

Year/Site		Overall
Age	< 25yr	140/231 (60.6%)
	25–30yr	77/231 (33.3%)
	>30yr	14/231 (6.1%)
Gender		Men 80/232 (34.5%)
Respondent is a parent		Yes 15/229 (6.6%)
Previous pediatric experience		Yes 104/232 (45%)
Response Rate	Saskatoon	232/338 (68.6%)
	Regina	

**Table 2:** Students’ attitudes towards the medical care of children

Item	Student Group	Pre Mean (SD)	Post Mean (SD)	t-test significance and effect size(d)
I will provide medical care to infants/children in my future medical practice	2nd year	4.18 (0.95)	4.22 (0.98)	*p* = 0.8 (*d* = −0.04)
	3rd year Saskatoon	3.89 (1.15)	3.76 (1.03)	*p* = 0.6 (*d* = 0.12)
	3rd year Regina	5.00 (0.00)	4.33 (1.21)	*p* = 0.3 (*d* = 0.78)
	ANOVA	*p* = 0.067	*p* = 0.056	
Care of children is valued within the medical profession	2nd year	4.61 (0.57)	4.33 (0.81)	*p* = 0.023 (*d* = 0.40)
	3rd year Saskatoon	4.04 (0.95)	4.24 (0.80)	*p* = 0.3 (*d* = −0.23)
	3rd year Regina	3.25 (1.71)	3.50 (1.38)	*p* = 0.8 (*d* = −0.16)
	ANOVA	*p* < 0.001*	*p* = 0.076	
Care of children is valued within society	2nd year	4.79 (0.45)	4.69 (0.57)	*p* = 0.3 (*d* = 0.21)
	3rd year Saskatoon	4.64 (0.53)	4.67 (0.48)	*p* = 0.5 (*d* = −0.06)
	3rd year Regina	4.75 (0.50)	4.33 (0.82)	*p* = 0.4 (*d* = 0.62)
	ANOVA	*p* = 0.3	*p* = 0.3	
Present income of physicians providing medical care to children is an incentive to consider providing medical care to infants/children	2nd year	2.06 (0.88)	2.10 (1.14)	*p* = 0.8 (*d* = −0.04)
	3rd year Saskatoon	2.20 (1.03)	2.05 (0.99)	*p* = 0.5 (*d* = 0.15)
	3rd year Regina	2.50 (1.00)	1.83 (0.98)	*p* = 0.4 (*d* = 0.68)
	ANOVA	*p* = 0.5	*p* = 0.8	
In the provision of care to well children, there is a significant role for nurse practitioners	2nd year	4.51 (0.61)	4.44 (0.79)	*p* = 0.6 (*d* = 0.10)
	3rd year Saskatoon	4.62 (0.49)	4.51 (0.55)	*p* = 0.5 (*d* = 0.21)
	3rd year Regina	5.00 (0.00)	4.67 (0.52)	*p* = 0.2 (*d* = 0.90)
	ANOVA	*p* = 0.2	*p* = 0.7	
In the provision of care to well children, there is a significant role for pediatricians	2nd year	4.49 (0.87)	4.59 (0.80)	*p* = 0.3 (*d* = −0.12)
	3rd year Saskatoon	4.47 (0.87)	4.64 (0.71)	*p* = 0.3 (*d* = −0.21)
	3rd year Regina	4.50 (0.58)	4.67 (0.52)	*p* = 0.8 (*d* = −0.31)
	ANOVA	*p* = 0.99	*p* = 0.92	
In the provision of care to well children, there is a significant role for family physicians	2nd year	4.70 (0.52)	4.83 (0.42)	*p* = 0.2 (*d* = −0.28)
	3rd year Saskatoon	4.73 (0.45)	4.76 (0.48)	*p* = 0.6 (*d* = −0.06)
	3rd year Regina	5.00 (0.00)	4.83 (0.41)	*p* = 0.4 (*d* = 0.57)
	ANOVA	*p* = 0.5	*p* = 0.7	
	Men		4.56 (0.78)	
	Women		4.88 (0.33)	
	t-test		*t*(49) = −2.45, *p* = 0.018, (*d*= −0.53)	

* Post hoc analysis: 2^nd^ year – 3^rd^ year Saskatoon; *p* = 0.001 (*d* = 0.73); 2^nd^ year – 3^rd^ year Regina; *p* = 0.003 (*d* = 1.07)

**Table 3:** Gender differences for 3^rd^ year students

Item	Student Group	Men Mean (SD)	Women Mean (SD)	t-test significance and effect size(d)
I will provide medical care to infants/children in my future medical practice	Pre	3.31 (1.11)	4.16 (1.12)	*p* = 0.022 (*d* = −0.76)
	Post	3.61 (0.92)	3.94 (1.17)	*p* = 0.292 (*d* = −0.31)
Care of children is valued within the medical profession	Pre	3.92 (1.04)	4.03 (1.04)	*p* = 0.758 (*d* = −0.11)
	Post	4.22 (0.88)	4.12 (0.93)	*p* = 0.707 (*d* = 0.11)
Care of children is valued within society	Pre	4.69 (0.48)	4.65 (0.54)	*p* = 0.797 (*d* = 0.27)
	Post	4.72 (0.46)	4.58 (0.56)	*p* = 0.349 (*d* = 0.27)
Present income of physicians providing medical care to children is an incentive to consider providing medical care to infants/children	Pre	2.62 (1.04)	2.11 (0.98)	*p* = 0.124 (*d* = 0.50)
	Post	2.17 (1.15)	1.94 (0.88)	*p* = 0.433 (*d* = 0.22)
In the provision of care to well children, there is a significant role for nurse practitioners	Pre	4.38 (0.51)	4.76 (0.44)	*p* = 0.014 (*d* = −0.80)
	Post	4.33 (0.59)	4.64 (0.49)	*p* = 0.056 (*d* = −0.57)
In the provision of care to well children, there is a significant role for pediatricians	Pre	4.46 (0.66)	4.49 (0.90)	*p* = 0.928 (*d* = −0.04)
	Post	4.61 (0.78)	4.67 (0.65)	*p* = 0.786 (*d* = −0.08)

## Appendix A

### Medical Care of Children Survey

Select the correct answers:

1. I am in:
  ___ Phase B
  ___ Phase C – Saskatoon
  ___ Phase C – Regina
2. Gender
  ___ Male
  ___ Female
3. Age
  ___ < 25 years
  ___ 25 – 30 years
  ___ >30 years
4. I am a parent:
  ___ Yes
  ___ No
5. Did you have previous clinical exposure to infant/child medical care before your Pediatrics Clinical Sciences session?
  ___Yes
  ___ No

If you answered no to question 5. Skip to question 7.

If you answered yes to question 5, please continue to question 6.

6. Previous clinical exposure in infant/child medical care (before Pediatrics Clinical Sciences sessions) included the following:
  ___ Shadowing
  ___ Switch/Search
  ___ SAHO summer externship
  ___ Community Experience
  ___ Other. Please list: ______________________________________
7. In previous clinical exposure, which health care professional(s) did you observe?
  ___ Pediatrician
  ___ Family Physician
  ___ Nurse Practitioner
  ___ Public Health Nurse
  ___ Other. Please list_________________________________________
8. List three (3) determinants of health important to Saskatchewan Children

Please rate the following statements:

**Table:**

	Highly unlikely	Unlikely	Neutral	Likely	Highly likely
9. I will provide medical care to infants/children in my future medical practice.					

**Table:**

	Highly disagree	Disagree	Neutral	Agree	Highly agree
10. Care of children is valued within the medical profession.					
11. Care of children is valued within society.					
12. Present income of physicians providing medical care to children is an incentive to consider providing medical care to infants/children.					

In the provision of care to well children, there is a significant role for:

**Table:**

	Highly disagree	Disagree	Neutral	Agree	Highly agree
13. Nurse Practitioners					
14. Pediatricians					
15. Family Physicians					

16. Where did your Pediatrics Clinical Sciences sessions take place? (Select all that apply):*
  ___ Hospital ward
  ___ Clinic/office
  ___ Other. Please list ___________________________________________________
17. Please share any comments/suggestions about your Pediatric Clinical Sciences Experience:*

## Appendix B

### Reflective Assignment Instructions

This is a one-two page (minimum 250 words; maximum 500 words) assignment reflecting on some of your learnings. Answer one or more of the following questions in the reflective assignment:

1. What skills do you feel are helpful for physicians in order to work effectively with children and their families? Describe these skills and why you feel they are important.
2. What determinants of child health did you observe as being important to some of the patients/families you encountered? Describe the impacts of one or two of these determinants of child health.
3. What were some of the common health issues or concerns of the pediatric patients/families you encountered? What health care providers are involved in the management of children’s health issues and what are their roles?

#### Due

Due by one week after the completion of the final session.
